# Insecticidal Effects of Plasma Treated Water

**DOI:** 10.3390/ijerph14121460

**Published:** 2017-11-27

**Authors:** Lars ten Bosch, Robert Köhler, Rinat Ortmann, Stephan Wieneke, Wolfgang Viöl

**Affiliations:** 1 Faculty of Natural Sciences and Technology, University of Applied Sciences and Arts, Von-Ossietzky-Strasse 99/100, 37085 Göttingen, Germany; robert.koehler@hawk-hhg.de (R.K.); rinat.ortmann@hawk-hhg.de (R.O.); stephan.wieneke@hawk-hhg.de (S.W.); wolfgang.vioel@hawk-hhg.de (W.V.); 2 Fraunhofer IST Application Centre, Von-Ossietzky-Strasse 100, 37085 Göttingen, Germany

**Keywords:** atmospheric pressure plasma, plasma, pest treatment, tap water, mealybug, *Planococcus citri*, plasma-based pest management

## Abstract

The efficacy of plasma-treated tap water (PTW) for the possible treatment of a mealybug (*Planococcus citri*) infestation was studied under laboratory conditions. Mealybugs growing on *Nerium oleander* have been treated using PTW after being transferred to Petri dishes, thus avoiding possible buffering effects that might occur in an in-situ study. When treating tap water with a dielectric barrier discharge for several minutes (1, 3, 5 and 10 min) a distinct acidification of the water can be determined, resulting in a pH value of 1.8 after 10 min treatment. The efficacies of the treated tap water samples were compared to the efficacies achieved using classically acidified water. The classical acidification of tap water was carried out using nitric acid and hydrochloric acid to see any possible influences of the salt of the acid in question. The application of PTW revealed high mortality rates of approx. 90% after an observation period of 24 h. PTW appears promising for the treatment of smaller plant stock and commodities as produced by small scale farmers or in greenhouses as an environmentally friendly substitute or supplement to conventional pesticides.

## 1. Introduction

Scale insects and other plant pest such as for example different aphids, infect cultivated plants [1], arable crops [2] as well as different tree species during all growing seasons [3]. Besides the direct impact on yield and quality, scale insects and aphids play a prominent role as vectors for other pests [4]. Their appearance is connected to closteroviridae infestations which are themselves affiliated with the little cherry disease [5], virus-associated grapevine leafroll [6] and rugose wood complex disease [7], and more. Common pests, occurring in almost every part of the world and in a vast variety of different genera are mealybugs. They belong to the *Pseudococcidae* family within the order of *Hemiptera*, which includes many pests, whose damage mainly involves impairment of the host plant due to sucking out the plant sap. Furthermore, mealybugs secrete large amounts of honeydew. These secretions are subsequently colonized by *Ascomycete* fungi, commonly referred to as sooty mold. Found on a variety of host plants, ranging from different coffee, vine, cotton and citrus plants, etc. mealybugs cause huge economic damage [8,9,10]. Their frequent occurrence, as well as their feeding behaviour on all plant parts, above ground as well as on root material depending on the genera, have made the mealybug a pest of interest for the pest treatment method presented here, as it offers promising applications both in the pest treatment as well as in the growth promotion field. 

Generally, common pest treatment methods range from chemical treatment to ecological and biological methods to physical approaches. Most chemical pest treatment remedies work with active substances which originate e.g., from the groups of organochlorides, organophosphates, carbamates, neonicotinoids, diamides, rotenoids a.m.m. or derivatives thereof [11,12,13]. These chemicals are applied to crops and cultivated plants in different forms (emulsions, dry powder, aerosols, fumigation). The possibility of coming into contact with these toxins during handling, mixing and application can cause various health hazards to users. 

Biological pest treatment methods focus mostly on the use of natural, non-chemical remedies. Different methods for natural pest regulation have been reported. One strategy, for example, aims at supporting existing species as well as the establishment of new predatory species [14,15] acting as natural enemies of specific pests. Furthermore, plants and plant parts prepared as pest control remedies [16] are able to contribute to a natural pest regulation, for example those used in Indian tea plantings [17]. 

Cultivation methods such as minimum or no-tillage approaches, inter-cropping [18] and companion planting [19] are also applied. Different concepts and the advantages thereof have been thoroughly covered within the agricultural science communities and are mostly treated under the umbrella of integrated pest management (IPM) and conservation biological control (CBC) covered by authors such as Horne and Page [20], and Barbosa [21] and others [22,23,24,25]. 

A relatively novel approach to control pests is based on a physical approach known as cold atmospheric pressure plasma (CAP). This plasma-based pest management (PBPM) is primarily used for the decontamination of food and feedstuffs. The applications range from the treatment of seeds [26] and seedlings [27] for the emergence of vigorous and healthy plants to degradation of food-borne pathogens [28,29] and secondary fungal metabolites [30,31]. Another aim is the plasma-based control of pest insects [32,33,34]. 

CAP’s have already shown their versatility within the young field known as plasma medicine. Different pathogens and diseases have shown a high susceptibility to the technology, which was therefore able to deliver therapeutic approaches over the last decade [35,36]. Especially within the fields of dermatology and oncology, diseases ranging from chronic venous leg [37] and ulcus [38] over the enhancement of epidermal microcirculation [39] to different tumor cells [40,41] were treated with success. The plasma medicine community is working with great efforts to understand and explain the occurring interaction mechanisms when applying plasmas in moist, biological environments. In addition, the fundamental insights [42,43] from this community will greatly contribute to the understanding of pesticidal and fertilization effects applicable within the field of plasma agriculture, as well. We would like to present here our findings on a novel insecticidal approach for PBPM. A feasibility study on the application of plasma-treated tap water (PTW) as insecticide was performed and the results are presented in the following sections.

## 2. Materials and Methods 

### 2.1. Preparation of Plasma Treated Liquids

**Figure 1:**
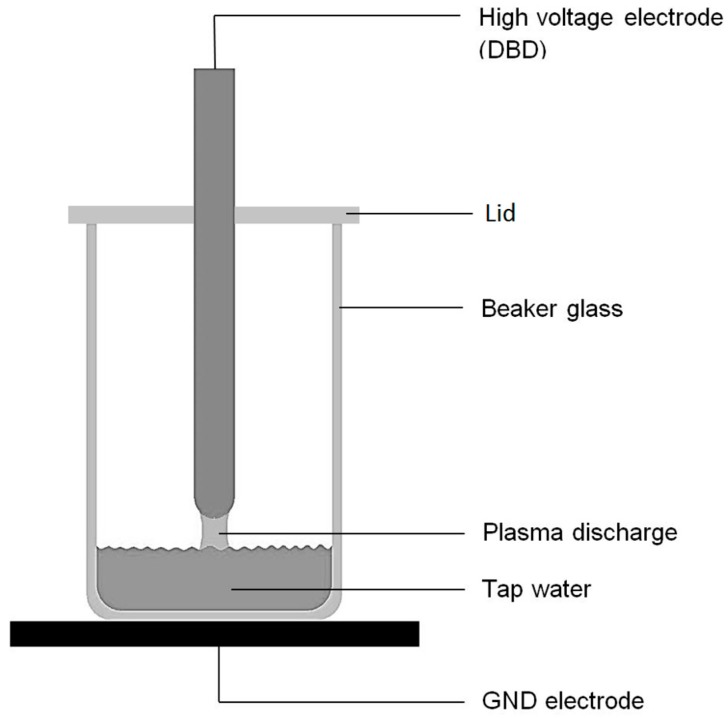
Experimental Setup for plasma treatment of regular tap water.

A dielectric barrier discharge was generated between a high voltage electrode and a water surface situated in a beaker glass in standard air as working gas (Figure 1). The high voltage electrode (HV-electrode) is composed of a copper powder core embedded in an Al_2_O_3_ ceramic. The ceramic is functioning as dielectric barrier (diameter ≈ 8 mm) producing a filamentary discharge at ambient pressure. High voltage was supplied via a power supply build in our laboratories giving the parameter set as displayed in Table 1. The high-voltage generation is based on the principle of a flyback converter. In addition to the usual design, a capacitor is located parallel to a switching transistor. Together with the primary winding of the high-voltage transformer, this setup forms a resonant circuit which is tuned to the resonance of a corresponding resonant circuit on the secondary side of the high-voltage transformer. The power is supplied via a 24 V DC-voltage source. The switching transistor is controlled by a microprocessor. The duty cycle of the transistor determines the amplitude of the generated high voltage. The switch-off time between two pulses is variable and selected in a way that ensures the remaining oscillation of the system to decay sufficiently, leaving the next high voltage pulse as unaffected as possible. Input parameter measurements were performed using a DLM2054 oscilloscope (Yokogawa, Musashino, Japan), a P6015A high voltage probe (Tektronix, Beaverton, OR, USA) and a Pearson current monitor model 2877 (Pearson Electronics, Palo Alto, CA, USA) to calculate the power input via U/I-method. The treatment times ranged from 1 min and 3 min to 5 min up to 10 min. To avoid losses, due to evaporation, a lid was applied to the beaker glass.

The HV-electrode was introduced into 20 mL beaker glass filled with 1 mL and 3 mL liquid, respectively. The effect of a plasma treatment was tested using standard tap water and deionised water. The whole setup was placed on a grounded aluminium plate. The electrode gap was fixed at distance of ≈3 mm above the liquid surface. No cooling of either the HV-electrode or the water sample was provided. The related temperature progression of the treated liquid quantities is displayed in the results Section 3.1. The temperature measurements were conducted using a UMI4 Universal Multichannel Instrument fibre optical temperature sensor utilising a FOT-L-SD-C1-F1-M2-R1-ST fibre optical sensor (both components from FISO Technologies Inc., Ville de Québec, QC, Canada). Small volumes of water were used to enable the quick production of water keeping the treatment times at a minimum. Comparing the volumes of 1 mL and 3 mL, a first impression concerning the dependece of the generation efficiency of different species with respect to the volume of water is given. The final experiments using PTW on mealybugs were performed using 1 mL of standard tap water.

### 2.2. Water Analysis

To avoid the use of liquids displaying particular requirements, the study focused on the use of regular tap water as pesticide. A comparison between regular tap water and deionised water was conducted to find possible deviations, when treated with a DBD. The analytical values of the tap water were provided by the Municipal Utilities of Göttingen, as published in Stadtwerke Göttingen AG [44]. Four parameters of the treated liquids were monitored (pH value, concentrations of nitrite, nitrate and hydrogen peroxide) as well as their development during the application of the air plasma before and during the experiments. The nitrate/nitrite and hydrogen peroxide measurements were performed using a reflectometer RQflex 10 (Merck Millipore, Darmstadt, Germany) applying tests no. 1.16971.0001 (nitrate); no. 116732 (nitrite) and no. 116974 (hydrogen peroxide). To compensate the crosstalk of the nitrate and nitrite during the tests, the liquids were diluted according to manufacturer specifications. Evaluation of pH values was conducted using a FiveEasy FE20 pH-electrode with measuring probe LE438 pμ (Mettler Toledo, Columbus, OH, USA). As the analytical measurements of the four parameters demand the temperature of the tested liquid to be max. 20 °C. All measurements were performed after a 5 min cooling phase following the respective treatment at ≈19 °C.

### 2.3. Classically Acidified Water 

A comparison of plasma-treated tap water (PTW) with two classically acidified waters (CAW) was conducted using hydrochloric acid and nitric acid (Th. Geyer GmbH & Co. KG, Renningen, Germany), respectively. This comparison serves two purposes; firstly, to visualize possible effects of PTW induced through plasma treatment other than mere acidification. Secondly, to discover possible influences of the respective salt of the acid in action. The pH values of the acidified water samples were adjusted according to the pH value of the produced PTW of ≈1.8. To avoid any side effects of possible tap water constituents the CAW were prepared using deionized water, using a Smart2Pure 3 UV/UF water purification system (Thermo Scientific, Barnstead, Waltham, MA, USA).

### 2.4. Test Organism Planococcus citri

Ex-situ tests were performed using mealybugs (*Planococcus citri*) to test the effectivity of the produced liquids as potential pesticide. The mealybugs were collected from greenhouses feeding on *Oleander nerium* (plants and organisms were kindly provided by the project partner W. Neudorff GmbH KG, Emmerthal, Germany). The specimens were bred under controlled conditions in a greenhouse setup, adjusting the lighting periods to 16 h/day at 20 °C. After carefully removing the individuals from the host plants, the vitality and mechanical integrity of each individual was assessed. The vitality tests were carefully performed using a binocular light microscope (Euromex Microscopen BV, Arnheim, The Netherlands) evaluating behaviour and torso movement response to tactile stimuli using bent tweezers (Manufactures D’Outils Dumont SA, Montignez, Switzerland).

To determine the pesticidal activity of PTW (positive control, n = 50), as well as of the two CAW (positive control, n = 100) the mealybugs were treated in an ex-situ environment using petri dishes (90 mm diameter × 15 mm height, Greiner Bio-One GmbH, Frickenhausen, Germany). Simultaneously, identically large negative controls were tested for mortality rates. Regular tap water functioned as a negative liquid control, as a non-pesticidal liquid to account for possible side effects, such a suffocation. Completely untreated mealybugs placed in a petri dish environment without any liquids being applied served as negative dry control. The ex-situ setup was chosen to avoid possible buffering or side effects originating from the habitat environment i.e., plants of *Oleander nerium*.

**Figure 2:**
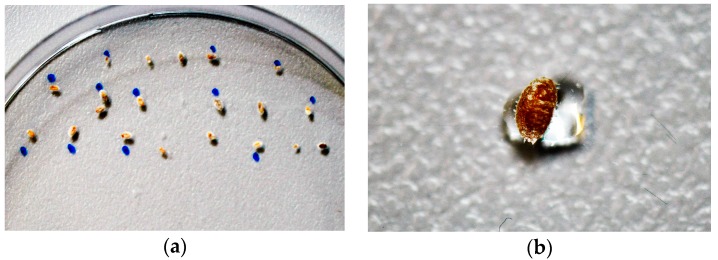
(**a**) Petri dish containing one test group of 20 mealybugs, each placed on one droplet (2 μL) of test liquid CAW; (**b**) Close-up of (**a**) single mealybug transferred to a test liquid droplet of PTW placed in a petri dish (droplet volume = 2 μL).

The petri dishes were prepared by applying one droplet of 2 μL liquid per individual onto the surface of each dish. The droplets were applied using an Eppendorf Research plus pipette with a working range of 0.1–2.5 μL (Eppendorf AG, Hamburg, Germany). Subsequently the mealybugs were transferred directly into the droplets, at a rate of one individual per droplet (see Figure 2a). This procedure was repeated for every test solution (PTW, CAW and TW). The dry reference samples were also transferred to a petri dish. All dishes were closed with their standard cover, no sealant applied, and stored at a room temperature of 20 °C for the duration of the experiment. A vitality assessment was carefully performed after 1 h, 3 h and 24 h, following the same procedure of tactile testing as described above. All consumable materials were purchased from Th. Geyer GmbH & Co. KG, Renningen, Germany.

## 3. Results

### 3.1. Analysis of PTW

**Figure 3:**
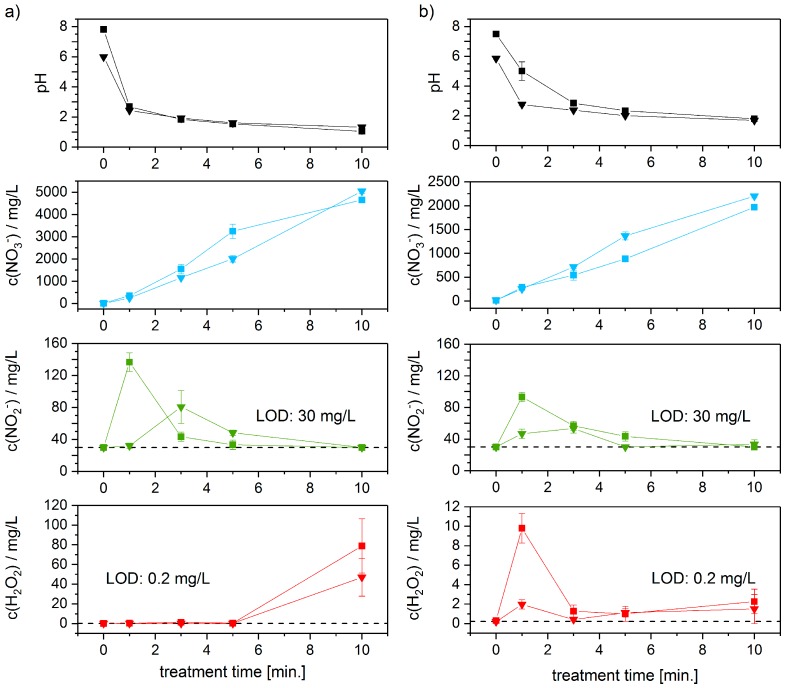
Development of concentration of pH, nitrate, nitrite and hydrogen peroxide due to CAP-treatment of (**a**) 1 mL treated liquid volume and (**b**) 3 mL treated liquid volume. The ■ data points show the behaviour of regular tap water (n = 3), whereas the ▼ data points depict the behaviour of deionized water (n = 3). Reducing the liquid volume from 3 mL to 1 mL the necessary treatment time to obtain comparable values of pH, nitrate, nitrite and hydrogen peroxide is reduced to 1/3, accordingly.

The generation of PTW, which was later applied to the mealybugs, was carried out by CAP treatment of regular tap water in air, for several minutes (setup see Figure 1). Part (a) of Figure 3 shows the behaviour of a liquid volume of 1 mL during the DBD treatment. Part (b) depicts the behaviour of 3 mL of liquid. The pH values, depicted in Figure 3, together with the values of NO_3_^−^ show the direct correlation between pH values and nitrate concentration of the liquid, as H^+^ and NO_3_^−^ ions must show equimolar distributions. H_2_O_2_ shows a notable increase after 5 min treatment time in the 1 mL liquid volume. The hydrogen peroxide in 3 mL liquid volume is rising immediately to decrease after 1 min treatment time, correlating with a pH value of less than 5. After this significant drop the H_2_O_2_ concentration rises again slowly after approx. 5 min of treatment time. 

The treated liquid volume of part (a) of Figure 3 is 1/3 of the liquid volume in part (b) of same figure. The development of the pH-value is correlating directly with the product of liquid volume and treatment time, i.e., similar pH-values are reached after identical volume treatment times (in min/mL). 

To effectively reduce the time for the conduction of the trials the following experiments were performed using liquid volumes of 1 mL, only. This measure reduced the necessary treatment time of TW to 3 min (compare Figure 3). The generation process is reliable and repeatable concerning the necessary treatment times per mL to obtain identical pH, nitrate, nitrite and hydrogen peroxide values (see standard deviation Figure 3; compare concentrations of 1 mL after 3 min and 3 mL after 10 min). Although, the hydrogen peroxide concentration is rising after 10 min treatment time to approx. 50 mg/L the following experiments were conducted using 1 mL TW treated for 3 min. Deionized water and regular tap water show an overall similar behaviour concerning the four substances under observation.

**Figure 4:**
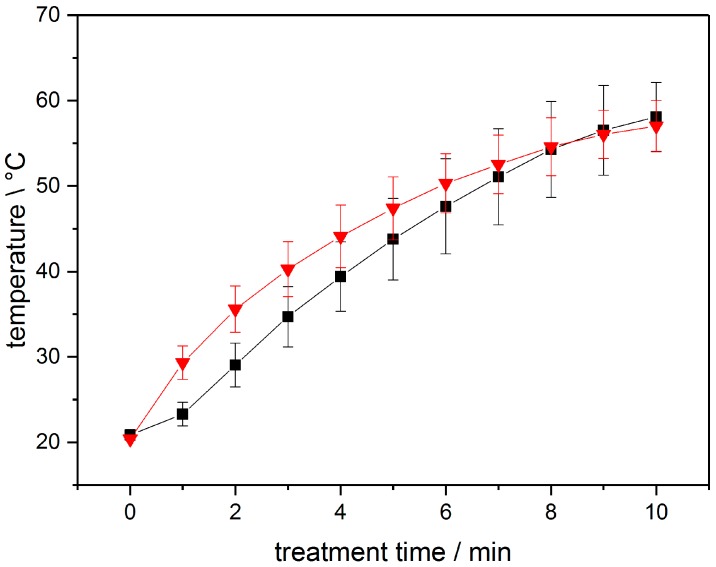
Temperature behaviour of 1 mL (red graph) and 3 mL (black graph) liquid during a plasma treatment using the setup displayed in Figure 1 (n = 3).

As mentioned in Section 2.1, the temperature during the treatment was evaluated using a fibre optical thermometer for online temperature monitoring. The temperatures behaviour of the different liquid volumes are displayed in Figure 4. No significant deviation of the temperature behaviour between tap water and deionized water could be measured. Therefore, Figure 4 displays the behaviour of the different tap water volumes, representatively.

Due to the more general availability of tap water, when thinking about larger applications of PTW, it was decided to conduct the following ex-situ experiments using TW for PTW preparation only.

### 3.2. PTW/CAW Treatment of Mealybugs

In the following, the mortality rates (MR) of mealybugs treated with the test liquids PTW and CAW are compared to the MR’s of different reference samples (dry and liquid references). During the experiment, it was observed that individuals treated with PTW or CAW showed almost no attempt of leaving the droplet areas, whereas the tap water treated samples and the dry reference samples did move around freely within the dish perimeters. 

**Figure 5:**
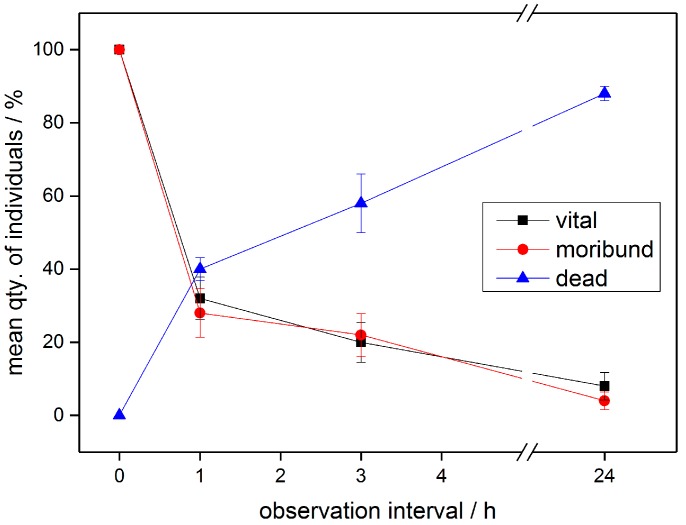
Development of the different vitality states vital, moribund and dead during the MR assessment after PTW treatment of mealybugs (*Planococcus citri*).

**Figure 6:**
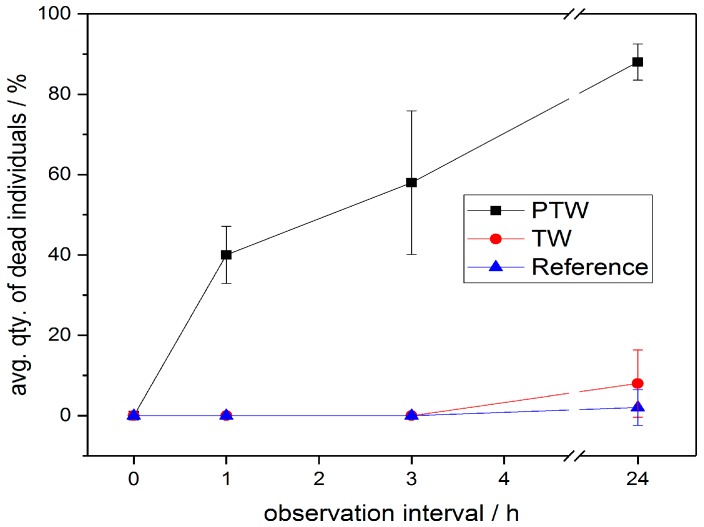
Comparison of mortification rates obtained using PTW and untreated TW vs. untreated reference samples. 3 replicates consisting of 10 individuals per treatment were used. The significance of differences between the treatment methods and the dry control were tested globally by one-way-ANOVA at α = 0.001 with correction for multiple testing, according to Bonferroni. The significant differences between PTW and TW are given with *p* = 6.82 × 10^−9^, (σ = 1). No significant differences between TW and dry reference were found.

Figure 5 displays three different vitality states of the treated insects during the PTW experiments. The insects rated to be in the vital state are showing immediate response to the tactile tests (see Section 2.4). When only showing retarded reaction to tactile testing, the insects are registered in the group of moribund individuals. The individuals that show no reaction to the tactile tests as well as no movement of extremities are assigned to dead group. Depicted in Figure 6 are the MR of the PTW treated samples compared to the reference samples that were treated with regular TW as well as compared to the dry reference samples.

**Figure 7:**
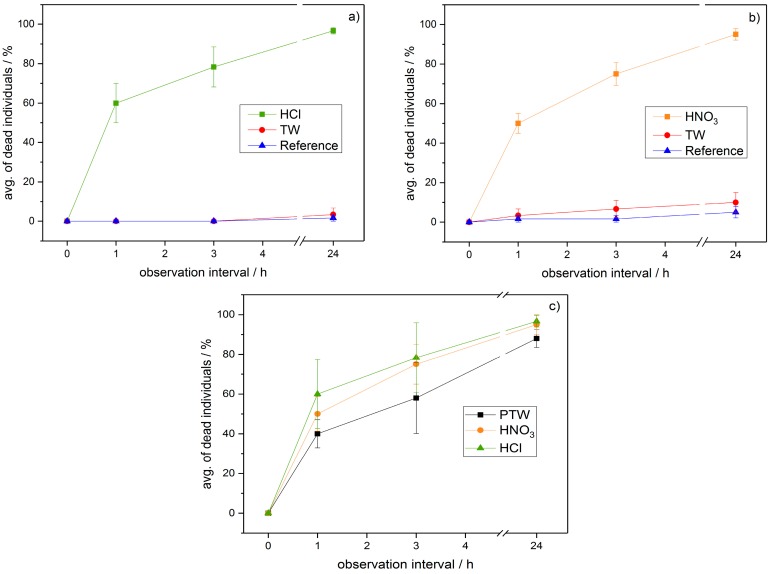
Mortification rates achieved using HCl (**a**) and HNO_3_ (**b**) compared to untreated reference samples as well as (**c**) direct comparison of the mortification rates of CAW/PTW; pH of 1.8 for all liquids tested. The significance of differences between the treatment methods were tested globally by one-way-ANOVA+Bonferroni, α = 0.001: (**a**) HCl/TW: *p* = 1.52 × 10^−6^, (σ = 1); (**b**) HNO_3_/TW *p* = 1.52 × 10^−6^, (σ = 1). (**c**) HCl/PTW: *p* = 0.008, (σ = 0), HNO_3_/PTW *p* = 0.02, (σ = 0); HNO_3_/HCl *p* = 1, (σ = 0).

As depicted in Figure 7a,b, the same general behaviour is found when CAW is applied to the mealybugs instead of PTW. When comparing the effectiveness of PTW and the two applied CAW, no significant differences can be stated (see Figure 7c). 

## 4. Discussion

Mealybugs, when treated by a single application of PTW ex-situ exhibit a MR of ≈85% after 24 h. When assessing the condition of the treated individuals a tactile test was performed similar to the testing stated in Section 2.4. The PTW treated mealybug samples show significantly higher mortality rates (MR) than the TW reference samples (MR ≈ 8%) and untreated reference samples (MR ≈ 2%) as displayed in Figure 6. Although the samples had been removed from their natural habitat, thus not being able to feed during the test period, the influence on the overall MR due to hostile environment is negligible. 

When comparing the effectiveness of PTW to the CAW the MR’s seem comparable; also, no significant differences between the respective salt of the acid in use (–Cl or NO_3_^−^) can be stated, as shown Figure 7c. As the pH values of the CAW were adapted precisely, according to the pH values of the PTW, the question arises why the effectiveness of PTW is lower than the effectiveness of CAW adjusted using nitric acid. It seems that other constituents of the PTW than nitric acid seem to have an influence on the mortality rate. An interesting candidate, able to further influence the effectivity of a plasma treated liquid when used as a pesticide, is hydrogen peroxide or OH radicals occurring during the generation pathway, respectively.

When applying a plasma discharge to a water surface the occurring hydroxyl radicals are offering a possible source for hydrogen peroxide. The effectivity of hydrogen peroxide formation through OH radical dimerization is depending on the applied parameter setting of each plasma source, as for example shown by Porter et al. [45] as well as Zhao et al. [46]. Examining the hydrogen peroxide concentration as depicted in Figure 3 is giving an idea of the responsible parameters. Comparing Figure 3 and Figure 4 give an impression of the influence of the pH-value of the solution as well as possible temperature influences on the hydrogen peroxide generation. When treating a small liquid volume, the formation of hydrogen peroxide seems to become most efficient when certain liquid temperatures are achieved. It is conceivable that the elevated temperatures are causing an increase in the humidity of the treating environment. This factor could very well promote the formation of OH and H_2_O_2_ within the volume of the humidified air at a much larger scale, compared to the standard liquid-Gas interface as apparent in lesser humid air. OH radical dimerization is acting as the major pathway for the formation of H_2_O_2_ in nitrogen and helium gases, as shown by Zhao et al. [46]. 

## 5. Conclusions

Although the mortality rates of CAW and PTW are almost identical (see Figure 7c), it should be kept in mind that the possibilities of this technology and its mode of actions should not be solely reduced to acidification of liquid media, as others have mentioned before [47]. The role of hydrogen peroxide as well as of the hydroxyl radicals must be examined thoroughly. First indications show that there may be an influence of the storage period between production and application concerning the effectiveness of the produced media. First tests using life plant material are giving first indications of the relation of shorter-lived species and the overall effectiveness of the applied media.

Furthermore, generation efficiencies of the occurring produced species are strongly dependent on application parameters such as temperature of working gas during treatment, average electron energy, working gas composition and humidity etc. Therefore, the ideal parameter settings and their interdependencies are currently under investigation. The use of CAP for PTW preparation can lead to the elimination of occupational hazards due to omission of handling and transport processes of harmful chemicals when utilised, e.g., for use in a greenhouse. Furthermore, the storage of potentially harmful chemicals can be avoided, as PTW is produced on demand. The technology can lead to the development of an efficient, environmentally friendly and safe pest treatment method.

Ongoing experiments of PTW and CAW applied to infested host plants show significant reduction of colony size of *Planococcus citri* without negatively effecting the host plants (*Oleander nerium*). Beneath the application of PTW as active pesticide first experiments give a great idea of the potential of this technology concerning growth mediation as well. The use of PTW as growth mediator solely as well as in combination with a liquid fertilizer [48], did reveal promising results as well as the direct plasma pre-treatment of seedlings [26].

To further develop this treatment method to a cost effectively alternative to standard procedures the energy efficiency of those plasma systems must be addressed and is part of ongoing studies.

## Figures and Tables

**Table 1:** Input parameters for experimental setup.

Input Parameter	Value
Electric power	≈11 W
Power density	≈5 W/cm^2^
Discharge gap	3 mm
Appl. voltage	≈30 kV (p-p)
Natural frequency	≈200 kHz
Puls rep. Rate	14 kHz
Waveform	decaying sine
